# Timeless and Stainless Alcohol: Concentric Waves from Its Oxidative Metabolism and Related Oxidative Stress

**DOI:** 10.3390/antiox15020216

**Published:** 2026-02-06

**Authors:** Riccardo Maccioni, Simone Tambaro, Laura Doro, Valentina Bassareo, Alessandra T. Peana, Elio Acquas

**Affiliations:** 1 Department of Immunology and Microbiology, The Scripps Research Institute, La Jolla, CA 92037, USA; rmaccioni@scripps.edu; 2 Department of Neurobiology, Care Sciences and Society (NVS), Division of Neurogeriatrics, Karolinska Institutet, 17164 Solna, Sweden; simone.tambaro@ki.se; 3 Department of Biomedical Sciences, University of Sassari, 07100 Sassari, Italy; l.doro@studenti.uniss.it; 4 Department of Biomedical Sciences, University of Cagliari, 09100 Cagliari, Italy; bassareo@unica.it; 5 Department of Medicine, Surgery and Pharmacy, University of Sassari, 07100 Sassari, Italy; apeana@uniss.it; 6 Department of Life and Environmental Sciences, University of Cagliari, 09100 Cagliari, Italy

**Keywords:** acetaldehyde, alcohol, ADH, ALDH, autophagy, catalase, CYP2E1, DNA methylation, inflammation, miRNA dysregulation, mitochondrial DNA, oxidative stress, reactive oxygen species, salsolinol

## Abstract

Alcohol is a molecule whose multiple effects in living organisms exemplify how profound biological complexity can arise from an exceptionally simple chemical structure interacting with the cellular biochemical machinery. This review was conceived to provide an up-to-date synthesis of the current knowledge on the multifaceted consequences of alcohol oxidative metabolism and alcohol-derived oxidative stress, ranging from disruption of subcellular and cellular homeostasis to impairment of organ function. This study primarily focuses on the consequences of alcohol metabolism and on the mechanisms by which the rise of its main metabolite, acetaldehyde, and of reactive oxygen species (ROS), generates oxidative stress by-products and molecular adducts responsible for compromising cellular energy balance and antioxidant defense mechanisms. In particular, this review aims to provide an exhaustive representation of the mechanisms, causes, and consequences of alcohol oxidative metabolism: this is accomplished by taking into account alcohol-induced modifications of gene expression of cellular antioxidant determinants, the role of epigenetic mechanisms, and that of gene polymorphisms linked to alcohol-dependent oxidative stress and responsible for serious diseases such as, among others, alcoholic hepatitis, cirrhosis, and hepatocellular carcinoma. In addition, this review highlights the role of alcohol oxidative metabolism in the brain, which, in the acute setting, activates the dopaminergic system mainly involved in alcohol reinforcing properties and, upon chronic exposure, contributes to neurodegenerative disorders. Finally, a dedicated paragraph explores autophagy as an integrative mechanism underlying the effects of alcohol-related oxidative stress across multiple organs, including the liver, heart, and brain.

## 1. Introduction

Because of its behaviorally reinforcing properties, alcohol is the most widely consumed pleasure-enhancing substance in the world [1]. However, due to chronic and heavy consumption, alcohol is also a significant threat to individual and public health, being directly and indirectly a risk factor for a number of serious and often irreversibly debilitating illnesses [2]. The present review encompasses an analysis of the literature, mainly preclinical, derived from a PubMed search focused on the last 15 years under the search keys “alcohol oxidative metabolism” and “alcohol-dependent oxidative stress (OS)”. This review focuses on alcohol oxidative metabolism as a central biological process influencing cellular functionality at multiple levels. Specifically, it examines how alcohol-induced OS disrupts energy production and antioxidant systems and how these alterations are associated with genetic and epigenetic modifications that further compromise the efficiency of cellular protective mechanisms. In addition, this review discusses how polymorphisms in genes encoding enzymes involved in alcohol oxidative metabolism modulate susceptibility to OS and its associated detrimental effects.

Alcohol is oxidized to acetaldehyde by alcohol dehydrogenase (ADH) and subsequently to acetate by aldehyde dehydrogenase (ALDH), both using NAD^+^ as a cofactor [3,4]. NAD^+^ is reduced to NADH, which, in turn, can be shunted to the mitochondrial electron transport chain (ETC). Thus, prolonged alcohol consumption may cause mitochondrial stress, which, together with an increased NADH/NAD^+^ ratio, leads to the generation of reactive oxygen species (ROS) [4]. Acetaldehyde forms adducts with lipids, proteins, and DNA [5], induces DNA mutations, and alters protein structure. Lipid peroxidation also plays an important role in alcohol-induced damage; the accumulation of some of its products, including malondialdehyde (MDA) and 4-hydroxynonenal, results in damage to DNA, proteins, and lipids [6]. Furthermore, the expression of the cytochrome P450 2E1 (CYP2E1), an isoform of another metabolic pathway involved in alcohol oxidative metabolism [7,8], the microsomal alcohol-oxidizing system (MEOS), is increased by chronic alcohol consumption. Such alcohol metabolism often results in uncoupled electron transfer, thereby directly producing superoxide anions and hydrogen peroxide rather than fully oxidized metabolites.

This review also discusses how, in the brain, alcohol may trigger the production of ROS and inflammatory factors, such as cytokines and NF-κB, and activate microglia and astrocytes, thereby transforming alcohol-dependent oxidative stress and chronic neuroinflammation into risk factors for neurodegenerative disorders, such as Alzheimer’s disease. In addition, this review also presents recent advances in peroxisomal catalase (CAT)-mediated oxidative metabolism of alcohol, which appears to be involved in the generation of the acetaldehyde-related adduct salsolinol. Recent experimental evidence indicates that salsolinol plays a key role in the alcohol-dependent activation of the mesolimbic dopamine system responsible for alcohol’s reinforcing properties. Finally, this review examines the role of autophagy as a cellular pathway involved in coordinating responses to alcohol-induced OS across different organs, with particular focus on the liver, heart, and brain.

Overall, as will be thoroughly discussed, chronic alcohol intoxication plays a critical role as a major inducer of OS [9]. In the whole body, and particularly in the brain, oxygen is extensively consumed and participates in numerous physiological processes [10], thereby making tissues vulnerable to OS [11]. Therefore, several antioxidant systems, both enzymatic and non-enzymatic, are present in the body [3], and particularly in the brain [10], to mitigate the potential damage induced by ROS [12], which exert destructive effects on cells [13]. However, while under physiological conditions ROS levels remain low [12], under intoxicating conditions they increase and accumulate because of an imbalance between reactive species and antioxidant processes [3]. This imbalance contributes to the onset and progression of different pathological conditions, including varying degrees of liver injury [8], cardiac and kidney failure [14], and neurodegenerative disorders [15]. Upon heavy alcohol drinking, these conditions are partially supported by the reduced expression of superoxide dismutase-1 (SOD1), glutathione peroxidase-1 (GPx1), and CAT genes, as well as by lower plasma SOD1 protein levels, together with increased OS biomarkers, such as thiobarbituric acid reactive substances, increased tumor necrosis factor-α (TNF-α), and reduced anti-inflammatory IL-10 [16].

Nevertheless, while this review adopts an OS-centered perspective, it is critical to acknowledge that parallel and complementary mechanisms—including the direct effects of alcohol, the involvement of immune signaling, and the role of gut endotoxins—also contribute to the biological consequences of alcohol and its oxidative metabolism.

## 2. Alcohol and Genetics: Understanding the Role of Oxidative Stress

### 2.1. Alcohol and Its Modulation of Antioxidant Defense Genes

Human studies and experimental animal models indicate that chronic alcohol consumption overwhelms cellular antioxidant systems and reprograms gene expression, thereby decreasing the transcriptional control of antioxidant and cytoprotective pathways [17]. This genomic dysregulation represents a major contributing factor to the persistent OS observed in alcohol-related tissue injury [18].

Evidence from animal models and in vitro studies supports the notion that a major molecular consequence of chronic alcohol exposure is the frequently observed reduction in the nuclear factor erythroid 2-related factor 2 (Nrf2) signaling cascade, the master regulator of cellular antioxidant defense [19]. Under normal conditions, Nrf2 is retained in the cytoplasm by its inhibitor, Kelch Like ECH Associated Protein 1 (Keap1) [20]. In response to OS, Nrf2 dissociates from Keap1, translocates to the nucleus, and binds to antioxidant response elements (AREs) in the promoter regions of detoxifying and antioxidant genes [21].

Preclinical studies indicate that chronic exposure to alcohol and its main metabolite, acetaldehyde, disrupts this adaptive mechanism [18]. Acetaldehyde forms protein adducts that prevent Nrf2 nuclear translocation or promote its proteasomal degradation, resulting in reduced transcriptional activation of ARE-dependent genes [22,23]. Reduced Nrf2 signaling has been associated with the downregulation of key antioxidant and detoxifying enzymes, including heme oxygenase-1 (HO-1), which mitigates OS and inflammation, NAD(P)H:quinone oxidoreductase 1 (NQO1), which detoxifies reactive quinones and prevents redox cycling, and glutamate-cysteine ligase (GCL), essential for maintaining intracellular redox balance [24]. Under conditions of OS, Nrf2 normally promotes the transcription of HO-1 and NQO1. HO-1 contributes to redox homeostasis by degrading pro-oxidant heme into biliverdin/bilirubin and carbon monoxide, which exert antioxidant and anti-inflammatory effects [25]. NQO1 reduces quinones to hydroquinones, thereby preventing redox cycling and limiting secondary ROS generation [26]. Impairment of Nrf2 signaling, therefore, diminishes these protective mechanisms, increasing cellular susceptibility to oxidative injury.

Reduced GCL transcription in rats, combined with excessive glutathione (GSH) utilization during the detoxification from ROS generated by CYP2E1 (see below Section 2.2) and other metabolic and inflammatory sources, leads to significant GSH depletion [27]. This depletion can impair the activity of glutathione peroxidase (GPx), which depends on GSH availability to detoxify hydrogen peroxide and lipid peroxides [28]. Consequently, cellular components become increasingly susceptible to oxidative damage, such as lipid peroxidation, protein oxidation, and mitochondrial DNA (mtDNA) injury (see below Section 2.3); all these features are commonly observed in chronic alcohol pathology in hepatic and extrahepatic tissues [29].

ROS can act as secondary messengers that activate the transcription factor NF-κB, resulting in its nuclear translocation and induction of pro-inflammatory cytokines, including TNF-α, IL-1β, and IL-6. In parallel, chronic alcohol intake enhances pro-inflammatory gene expression, further amplifying oxidative injury [30]. This self-perpetuating cycle of inflammation and OS maintains tissue injury and contributes to the development of alcohol-related diseases, particularly alcoholic liver disease, as well as other diseases affecting multiple organs [30].

Taken together, alcohol-induced OS emerges as one component of a broader pathogenic network arising from excessive ROS production, suppression of antioxidant defense mechanisms, and enhanced pro-inflammatory signaling. Notably, the interplay between impaired Nrf2-regulated pathways, GSH depletion, NF-κB activation, and parallel non-oxidative mechanisms, rather than any single pathway acting in isolation, may collectively contribute to alcohol-induced organ toxicity [31].

### 2.2. ADH and ALDH2 Polymorphisms and CYP2E1 Variants: Role in ROS Accumulation and in Increased Vulnerability to Oxidative Injury

Human genetic association studies demonstrate that susceptibility to alcohol-induced oxidative injury and diseases varies considerably among individuals and is primarily determined by functional genetic polymorphisms in key alcohol-metabolizing enzymes: ADH, ALDH2, and CYP2E1 [32]. These genetic variants alter alcohol oxidation rates, acetaldehyde accumulation, and ROS production, thereby modulating cellular vulnerability to OS and tissue damage in concert with environmental and metabolic factors.

Genetic variation significantly affects the initial steps of alcohol metabolism, specifically the oxidation of alcohol to acetaldehyde by ADH, as well as the subsequent conversion of acetaldehyde to acetate by ALDH2. This effect is especially pronounced in East Asian populations, where specific polymorphisms can both increase acetaldehyde production and reduce its detoxification, creating a toxic metabolic environment strongly associated with oxidative injury [33].

Variants at the ADH1B locus encode enzymes with distinct catalytic efficiencies. The ADH1B*2 allele produces an enzyme with a much higher catalytic rate than the wild-type ADH1B*1, resulting in rapid acetaldehyde accumulation following alcohol intake. Acetaldehyde then forms DNA and protein adducts, which are associated with increased OS and cellular stress. In individuals who persist in heavy alcohol consumption despite the adverse effects of high acetaldehyde levels (flush reaction), these surges are linked to an elevated risk of cancer, particularly oesophageal squamous cell carcinoma [34].

**Figure 1:**
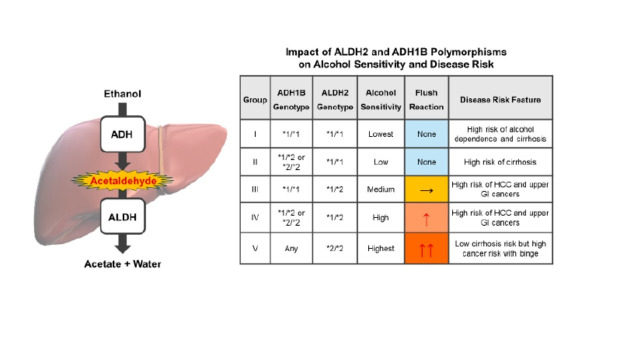
Genetic polymorphisms in alcohol metabolism and their clinical implications. The table classifies individuals into five groups based on their ADH1B and ALDH2 genotypes, alcohol sensitivity, and flushing response. These groupings correlate with specific clinical risks, such as alcohol dependence, cirrhosis, and upper gastrointestinal cancers. Alcohol-induced hepatic damage is mediated by the direct hepatotoxic effects of alcohol, the accumulation of acetaldehyde, and the disruption of the gut barrier. **→** represents a moderate flushing response, **↑** represents a severe flushing response, and **↑↑** represents a very severe flushing response. Reprinted/adapted with permission from Ref. [33]. 2025, T. Tadokoro.

ALDH2 variants, which are decisive in regulating acetaldehyde clearance, are equally critical for increasing vulnerability to alcohol metabolism-mediated toxicity. The ALDH2*2 allele (Glu487Lys substitution) markedly reduces enzymatic activity by impairing its affinity for the NAD^+^ cofactor, resulting in diminished acetaldehyde detoxification [35]. Individuals homozygous for this allele, after alcohol consumption, accumulate high levels of acetaldehyde, which cause flushing, tachycardia, and nausea (Figure 1).

Human observational data, supported by experimental evidence, indicate that prolonged exposure to acetaldehyde is associated with chronic OS, mitochondrial dysfunction, and activation of inflammatory pathways [33,35,36]. Consequently, individuals carrying the ALDH2*2 allele who continue to drink alcohol face a significantly higher risk of alcohol-related cancers, cardiovascular injury, and other oxidative pathologies [36] (see also below Section 4.1 and Section 4.2).

While ADH and ALDH2 polymorphisms influence the exposure to oxidative injury primarily through acetaldehyde metabolism, CYP2E1 variants have a more direct impact by modulating intracellular ROS production. CYP2E1, a key component of the MEOS, metabolizes alcohol predominantly upon chronic exposure. Its catalytic process is inefficient, often resulting in uncoupled electron transfer that produces superoxide anions and hydrogen peroxide rather than fully oxidized metabolites [37]. This excess **of** ROS may also contribute to lipid peroxidation, protein oxidation, and mtDNA damage.

Genetic variants, such as the CYP2E1*5B (c2) allele located in the gene’s regulatory region, are linked to higher basal transcriptional activity and increased inducibility [33]. Individuals carrying such high-activity alleles exhibit elevated CYP2E1 expression in response to alcohol exposure, leading to greater ROS production and heightened susceptibility to oxidative tissue injury, particularly in the liver [38]. Thus, CYP2E1 polymorphisms may establish a direct genetic connection between alcohol metabolism and ROS-mediated hepatotoxicity, as supported mainly by animal and mechanistic studies [38].

The greatest susceptibility to alcohol-induced oxidative injury occurs in individuals with combined polymorphisms that simultaneously increase acetaldehyde and ROS accumulation. Those possessing a fast-acting ADH1B*2 variant (i.e., rapid acetaldehyde production), a slow-activity or inactive ALDH2*2 variant (i.e., impaired acetaldehyde clearance) [33], and a high-activity CYP2E1 allele (i.e., increased ROS generation) experience a synergistic metabolic imbalance. This synergy increases overall cellular stress, promoting mitochondrial dysfunction, inflammation, and thereby increasing disease risk, including liver cirrhosis, cardiomyopathy, gastrointestinal cancers, and neurodegeneration [39] (see below Section 4 and Section 5).

Elucidating these gene–environment interactions is essential for understanding interindividual variability in alcohol-induced oxidative damage and identifying opportunities for personalized prevention and therapeutic strategies. Approaches that target CYP2E1 activity or that pharmacologically reduce acetaldehyde-induced OS may be particularly beneficial for genetically susceptible populations.

### 2.3. Alcohol, Its Oxidative Metabolism, and Mitochondrial DNA

Alcohol consumption, especially chronic or binge drinking, is strongly associated with mitochondrial OS and mtDNA damage in mice [40]. These effects reflect the convergence of oxidative, metabolic, and inflammatory stressors, rather than mitochondrial ROS acting alone.

Experimental studies demonstrate that alcohol metabolism, particularly via the hepatic enzyme CYP2E1, plays a central role in generating ROS and in disrupting the cellular redox balance through such excessive production. Mitochondria, as both a source and a target of ROS, are particularly vulnerable. ROS can induce mtDNA lesions, such as 8-hydroxydeoxyguanosine accumulation, whereby single- and double-strand breaks lead to mitochondrial dysfunction [41].

Alcohol exposure has also been shown, in both animal and in vitro models, to impair mtDNA repair enzymes, including 8-oxoguanine DNA glycosylase-1, which is responsible for repairing oxidative lesions [42]. Impaired repair capacity causes mtDNA damage to accumulate over time. Acetaldehyde can directly form covalent DNA adducts, including those involving mitochondrial DNA. In parallel, acetaldehyde-mediated adduct formation and oxidative modification of mitochondrial topoisomerases impair their activity, thereby compromising mtDNA replication and maintenance.

Chronic alcohol consumption also suppresses the synthesis of mtDNA-encoded protein subunits of ETC complexes I, III, IV, and V, compromising mitochondrial bioenergetics [43]. Damaged or depleted mtDNA leads to reduced ATP production and altered membrane potential, thereby impairing cellular energy homeostasis [44]. As primarily shown in experimental systems, damaged mtDNA can also be released into the cytoplasm or into the extracellular space (via exosomes), where it acts as a damage-associated molecular pattern, activating inflammatory signaling pathways such as cGAS-STING, a signaling pathway key mediator of inflammation in both peripheral and central systems [45], cellular stress, tissue damage, and TLR9 receptors activation [46].

Based largely on preclinical evidence, these persistent conditions of mitochondrial dysfunction and inflammation contribute to the pathogenesis of alcohol-related diseases such as alcoholic liver diseases, cardiomyopathy, and neurodegeneration [46] (see also below Section 4 and Section 5). In summary, alcohol profoundly impacts mtDNA integrity and quantity, sustaining a feed-forward cycle of OS, mitochondrial dysfunction, and inflammation that underlies the multi-organ pathology associated with chronic alcohol consumption (see also below Section 6 on the role of autophagy in this integrated process).

## 3. Alcohol and Epigenetics: Understanding the Role of Oxidative Stress

The term “epigenetics” refers to the ensemble of molecular mechanisms that induce changes in gene expression without altering the underlying DNA sequence [47]. DNA methylation [48], histone post-translational modifications [49], and the regulatory activity of non-coding RNAs, particularly microRNAs [50], are among the canonical epigenetic processes which, together, shape chromatin architecture and transcriptional output. More recently, RNA chemical modifications, especially RNA methylation [51], have been recognized as an additional layer of epigenetic control, expanding the concept of the “epigenome” to include that of the “epitranscriptome” [52]. The interplay between OS and epigenetic regulation has emerged as a critical determinant of cellular homeostasis and disease progression [53]. This section explores the relationship between alcohol-induced OS and the epigenetic and epitranscriptomic landscape.

### 3.1. Alcohol-Induced Aberrant DNA Methylation and Histone Acetylation and Their Role in Oxidative Stress

DNA methylation is a major epigenetic mechanism governing transcriptional activity, genomic stability, and cellular identity [48]. It is catalyzed by DNA methyltransferases (DNMTs) enzymes that transfer methyl groups from the methionine-derived coenzyme S-adenyl methionine (SAM) to the 5’ carbon of a cytosine residue in CpG dinucleotides [48]. This process determines a stable but reversible layer of transcriptional regulation that can be dynamically altered by multiple factors, including alcohol exposure. Accordingly, several epidemiological studies have reported changes in the DNA methylation profile of individuals who consume alcohol [54,55,56,57], and, accordingly, DNA methylation biomarkers have been proposed as novel and reliable measures of alcohol use [58]. DNA methylation and its regulatory mechanisms are heavily involved in alcohol-induced neurodevelopmental toxicity [59,60], synaptic plasticity [61], as well as in alcohol sensitivity and drinking behavior [61,62,63].

Due to its disruptive effects on the one-carbon metabolism network [64], alcohol has often been associated with reduced levels of the universal methyl donor SAM, while increasing S-adenosylhomocysteine (SAH), a potent inhibitor of DNMTs [65,66,67]. The consequent reduction in the SAM/SAH ratio diminishes global methylation potential, consistent with reports of decreased DNMT1 and DNMT3a/b expression and DNA methylation in animal models and in patients with alcohol use disorder (AUD) [57,68]. Conversely, other studies on animal models have reported upregulation of DNMTs and DNA methylation after chronic alcohol exposure [69]. Thus, alcohol-induced DNA methylation changes appear to be bidirectional and context-dependent, varying across genes, tissues, and disease stages [70].

OS might constitute a key mediator in alcohol-induced perturbation of DNA methylation, and changes in DNA methylation arising from OS are mainly due to alterations in DNMT activity. Both methionine adenosyltransferase (MAT), the enzyme that converts methionine into SAM, and DNMT1 have redox-sensitive residues in their catalytic sites and can be inhibited by oxidative imbalance [71]. Moreover, ROS can exert direct oxidative damage on DNA bases, leading to the oxidation of methylated cytosine or guanine within the CpG dinucleotide [72,73]. Oxidation of methylated cytosine bases produces 5-Hydroxymethylcytosine, and elevated cellular levels of 5-Hydroxymethylcytosine may promote DNA demethylation [72]; on the other hand, guanine is oxidized to 8-oxoguanine, resulting in the reduction of the binding affinity to DNMTs, thereby preventing the methylation of CpG islands [73].

Histone post-translational modifications are another example of covalent yet reversible chemical changes that dictate chromatin accessibility and transcriptional competence [49]. Similarly to DNA methylation, histone epigenetic marks are regulated by a network of enzymes that “write” or “erase” the marks (e.g., histone acetyltransferases, HATs, and histone deacetylases, HDACs) [74]. Histone acetylation is among the most studied alcohol-induced post-translational modifications: this has been associated with alcohol’s toxic manifestations in the liver, a mechanism that might be tightly interconnected with OS. In fact, in both hepatocytes and in rat liver, alcohol increases histone H3 acetylation at lysine 9 (H3K9ac) at the promoter of the ADH1 gene [75]. This localized hyperacetylation results in the transcriptional upregulation of ADH1 [75] and, consequently, in the upregulation of ADH1-mediated oxidative alcohol metabolism and its derivative toxic metabolite and ROS. This could trigger a maladaptive epigenetic stress response, creating a loop that perpetuates histone acetylation and OS. In fact, OS is not solely a consequence, but also a cause of histone acetylation [76]. In agreement, it has been shown that pretreatment with antioxidants reduces alcohol-induced H3K9ac and ADH1 levels in rat hepatocytes, while ROS inducers boost both alcohol-induced H3K9ac and ADH1 levels [76]. Finally, as already mentioned, alcohol metabolism increases NADH and depletes NAD^+^: this leads to inhibition of the NAD^+^-dependent deacetylase SIRT1 and promotion of histone hyperacetylation [77]. SIRT1 is a master regulator of antioxidant defenses, and its inhibition promotes inflammatory gene expression, further ROS accumulation, and exacerbation of liver damage [77,78].

### 3.2. miRNA Dysregulation, RNA Methylation, and Alcohol-Induced Oxidative Stress

MicroRNAs (miRNAs) are small non-coding RNAs that conventionally regulate gene expression at the post-transcriptional level by promoting mRNA degradation or inhibiting translation [79]. Acting as a flexible and reversible layer of regulation without altering the DNA sequence, miRNAs are integral components of the epigenetic landscape [50]. Alcohol exposure and alcohol-derived OS profoundly alter miRNA expression across multiple tissues and organs [80]. These alterations can result from direct alcohol-induced damage to enzymes essential for miRNA biogenesis, such as Dicer and Drosha [81], or may also derive from the redox-sensitive activation of transcription factors such as NF-κB [82,83]. Among alcohol-sensitive miRNAs, several directly modulate OS. In human and rat liver cells, miR-214 is upregulated after chronic alcohol exposure in both human cells and alcohol-fed rats and targets GSH reductase and P450 oxidoreductase, thereby depleting reduced GSH and weakening antioxidant defenses while enhancing ROS production [84]. miR-155, another pro-inflammatory miRNA, is increased by alcohol in rodents and promotes neuroinflammation by stimulating TNFα and monocyte chemoattractant protein-1 (MCP1) [85] and liver fibrosis through MCP1 and peroxisome proliferator-activated receptor alpha (PPARα) [86]. miR-21 is also elevated in the injured liver of heavy alcohol consumers and targets the von Hippel-Lindau gene, enhancing NF-κB-dependent inflammatory signaling in murine hepatic stellate cells [87], further exacerbating oxidative damage and miRNA dysregulation, as discussed above. In contrast, miR-223 plays a protective role in alcohol-induced liver disease, as it is upregulated in the serum and neutrophils of alcohol-fed mice and directly inhibits IL-6 expression, blocking neutrophil infiltration and ROS production [88]. Collectively, alcohol-induced miRNA dysregulation integrates inflammatory and epigenetic pathways into a unified pathogenic axis, whereby OS alters miRNA networks that, in turn, amplify redox imbalance and tissue damage.

RNA modifications constitute a critical post-transcriptional regulatory layer that complements other canonical epigenetic mechanisms. More than 170 distinct chemical marks have been identified across coding and non-coding RNAs, regulating their splicing, stability, localization, and translational efficiency [52]. Among these, RNA methylation, particularly N6-methyladenosine (m6A), represents the most abundant and dynamically regulated modification [51]. RNA methylation is catalyzed by enzymes that add (e.g., METTL3), remove (e.g., Fat mass and obesity-associated protein, FTO), and read (e.g., YT521-B homology domain containing family, YTHDF) methyl groups to the RNA [51]. Recent evidence suggests that alcohol dependence induces robust m6A hypermethylation in the hippocampus of mice in acute withdrawal and that neuronal deletion of the m6A eraser, FTO, enhances the motivational response to alcohol, accelerates the onset of dependence, and increases the risk of relapse drinking [89]. Aberrant RNA methylation status has also been reported in the nucleus Accumbens of postmortem subjects with AUD [90] and in the stop codon of opioid receptor genes in mice after chronic alcohol intermittent exposure [91]. However, the interplay between alcohol-induced alterations in RNA methylation patterns and OS has been less explored than for other epigenetic regulatory mechanisms.

Similarly to DNA methylation, RNA methylation requires the universal methyl donor SAM [92]. Therefore, alterations in one-carbon metabolism and the SAM/SAH ratio by alcohol-induced OS might also be a shared mechanism by which alcohol dysregulates both DNA and RNA methylation. In murine hepatic tissue, chronic alcohol intake induces the degradation of the m6A methyltransferases METTL3 [93]. This degradation disrupts m6A modification in the liver, leading to Activating Transcription Factor overactivation and increased neutrophil infiltration and inflammatory cytokine production [93]. Finally, in vitro and in vivo studies in mice show that alcohol increases the m6A methylation of PPARα by FTO-mediated YTHDF2 epigenetic modifications, leading to the activation of NLR family pyrin domain containing 3 (NLRP3) inflammasomes and NF-κB-driven OS and renal inflammation [94].

Collectively, these observations suggest a role of an OS-epigenetic axis in governing aspects of alcohol toxicity and, although supported by limited evidence, also its motivational properties. Alcohol-induced OS might function as a central integrator of epigenetic dysregulation across multiple regulatory layers, and its bidirectional interplay with the epigenetic landscape may represent an emerging mechanism by which alcohol drives molecular changes leading to deleterious pro-inflammatory responses and self-sustaining pathological loops across multiple tissues and organs.

## 4. Role of Oxidative Metabolism and Oxidative Stress in the Peripheral Effects of Alcohol

### 4.1. Alcohol-Induced Oxidative Stress and Its Impact on Liver and Gut

As a consequence of heavy alcohol consumption, steatosis, severe jaundice, fibrosis, cirrhosis, and hepatocellular carcinoma (HCC) appear as interconnected components of a clinical spectrum characterizing the most severe manifestations of alcohol-associated hepatitis (AH) and alcoholic liver disease (ALD) [95,96].

The alcoholic liver, in response to excessive alcohol consumption, undergoes a metabolic derangement rooted in compromised lipid metabolism; this impairment is characterized by abnormal esterification of cholesterol, excessive synthesis of triglycerides, and elevated LDL levels [97]. Moreover, alcohol-dependent availability of acetaldehyde, as well as other aldehydes generated by lipid peroxidation and OS, induces collagen synthesis and the generation of protein and DNA adducts, which trigger both TGFβ-dependent and TGFβ-independent pro-fibrogenic pathways in activated stellate cells [98]. This activation significantly increases extracellular matrix production [99] and plays a critical role in the pathogenesis of AH [100]. In addition, acetaldehyde alters lipid homeostasis by decreasing peroxisome proliferator-activated receptor (PPAR) expression [101] and, by increasing sterol regulatory element binding protein activity via an AMP-activated protein kinase (AMPK)-dependent mechanism [102], promotes lipogenic gene expression and hepatic triglyceride accumulation [103].

Oxidative metabolism of alcohol via ADH- and ALDH-mediated reactions also leads to NADH accumulation and to a consequent increase in the NADH/NAD^+^ ratio (as discussed above in Section 2.1 and Section 2.2) with significant effects on key biochemical pathways such as glycolysis, citric acid cycle, fatty acid oxidation, and glucogenesis. NADH is mainly oxidized to NAD^+^ by the mitochondrial ETC [104,105], a process that transfers electrons to oxygen and generates ROS such as superoxide anion (O_2_^•−^), hydrogen peroxide (H_2_O_2_), and the hydroxyl radical (HO^•^) [104,105].

SOD, CAT, GPx, and GSH possess strong free radical–scavenging capabilities and act as a critical antioxidant shield in the liver. In particular, GSH is essential for maintaining intracellular redox homeostasis [106] and, as a ubiquitous and abundant non-enzymatic antioxidant, acts as a key determinant of tissue susceptibility to oxidative damage throughout the entire organism [107]. Accordingly, mice and rats chronically administered alcohol exhibit significantly reduced hepatic levels of SOD, CAT, GPx, and GSH [108,109] (see also above Section 2.1). This finding indicates that antioxidant enzymes and GSH are consumed while counteracting oxidative damage and maintaining redox homeostasis. In this context, it is not surprising that N-acetylcysteine (NAC), acting as a cysteine donor and GSH precursor, effectively restores GSH availability. Notably, given that corticosteroids represent the gold standard therapy for AH-associated inflammation [110], NAC was reported to significantly reduce mortality rates when combined with prednisolone for the management of AH in a human observational study [111]. Indeed, acetaldehyde may also form adducts responsible for activating innate immune responses. These, together with products of mitochondrial damage, sensitize hepatocytes to other insults such as inflammatory factors. In this regard, the inflammatory responses activated in ALD share a common role in OS but also in innate and adaptive immune activation, where OS initiates alcohol metabolism-dependent hepatocellular injury. In this context, immune and inflammatory responses are crucial determinants for the progression into more severe clinical conditions.

Alcohol consumption is known to damage gut integrity and homeostasis [112], exerting both direct and indirect negative effects on alcohol-related liver pathophysiology. In fact, gut microbiota is also recognized as a critical player in the development of AH and ALD [112], as disruption of gut microbiota functional integrity by excessive alcohol consumption leads to alterations in the gut–liver axis microbiome [113] and increases the permeability of intestinal epithelial cells [114]. Consequently, bacterial-derived antigens migrating to the liver trigger inflammation, hepatocyte death, and liver fibrosis [114]. The role of the gut–liver axis remains pivotal in the liver pathophysiology since gut microbiome imbalance also directly activates pathways of liver OS. Accordingly, the condition of sterile inflammation, determined alongside alcohol and ROS by different factors such as xenobiotics, which are endogenous compounds responsible for cholestatic obstruction or free fatty acids, rapidly recruits neutrophils and the release of NETs (Neutrophil Extracellular Traps) as part of the inflammatory cascade. This contributes to the progression of liver damage by recruiting hepatic stellate cells responsible for hepatic fibrosis upon trans-differentiation into extracellular matrix proteins secretory myofibroblasts. Accordingly, targeting the gut microbiota has emerged in recent years as a promising therapeutic strategy for AH [115]. Thus, several clinical trials have investigated the effect of gut sterilization through antibiotics, either given alone or in association with glucocorticoid therapy. This strategy was aimed at reducing inflammatory factors such as NF-κB [116], pro-inflammatory cytokines (IL-1 and IL-8), and TNFα [117]. Accordingly, the addition of rifaximin to glucocorticoids over 90 days of treatment revealed a significant reduction of complication events, although ineffective on mortality incidence [118]. On the same vein, in a randomized clinical study, the treatment of patients with AH with the association of amoxicillin and clavulanate combined with prednisolone resulted in a lack of survival improvement in comparison to prednisolone alone [119].

Overall, since a number of factors may be recognized as critical determinants of dysbiosis, including diet [120], altered circadian rhythms [121], chronic stress [122], and alcoholic beverages [123], the mechanism by which alcohol consumption may promote gut impairment and contribute to compromise its integrity [124] can be fully understood by recognizing the physiological role of the gut–liver axis integrity and the role played by such critical determinants.

### 4.2. Alcohol-Induced Oxidative Stress as a Common Denominator Underlying Cardiac, Renal, and Pulmonary Disorders

Prolonged and heavy alcohol intake may disrupt redox balance in cardiomyocytes and promote excessive ROS production responsible for lipid, protein, and DNA oxidation in these cells, leading to OS-induced cardiac apoptosis [125]. Acetaldehyde plays an important role in myocardial injury by altering actin–myosin interaction and inducing mitochondrial dysfunction [125]. Moreover, as previously discussed (see above Section 2.2), ALDH2 could be inactivated by polymorphisms, thus leading to an augmented risk of cardiovascular diseases such as coronary artery syndrome, alcohol-induced cardiac dysfunction, pulmonary arterial hypertension, and heart failure [126]. From Hu and colleagues’ studies (2022), it was shown that moderate alcohol consumption in heterozygous mice could damage cardiac function, probably by augmenting ROS [127]. Consequently, individuals with ALDH2 polymorphisms should avoid alcohol drinking to reduce the probability of cardiovascular impairment caused by even such moderate ingestion [127]. Nevertheless, moderate alcohol consumption in otherwise healthy individuals has been reported as protective against certain cardiovascular diseases [128].

Acute alcohol consumption negatively affects the heart, inducing acute alcoholic cardiomyopathy, which is defined as alcohol toxicity to the heart muscle itself by alcohol and its metabolites. Abundant evidence supports that the mitochondrial dysfunction-induced myocardial OS plays a critical role in the subcellular pathogenesis of alcoholic cardiomyopathy [129,130]. This can be characterized by distinguishing between acute and chronic forms based on the initiation time and on the pathological characteristics. Both forms are characterized by myocardial inflammation, reduced cardiac cell contraction, heart failure, arrhythmias, and left ventricular dilation [131], with the chronic form being a leading cause of non-ischemic dilated cardiomyopathy.

Different studies have established that alcohol induced mitochondrial damage contributes to myocardial OS, as well as to the subsequent development of acute alcoholic cardiomyopathy. Similarly, damaged mtDNA, released by exosomes, disrupts lung epithelial barrier integrity and impairs macrophage phagocytic function, thus increasing susceptibility to respiratory infections [132]. Postmortem results from patients with alcoholic cardiomyopathy disclose evident mitochondrial alterations and augmented lysosome numbers [133]. In vivo and in vitro experimental evidence further corroborates this observation, indicating that acute alcohol consumption and exposure lead to important cardiomyocyte mitochondrial pathologies, characterized by alterations in the mitochondrial membrane, reduced membrane potential, and changed ATP production [129,130], as well as in autophagy downregulation [129] (see also below Section 6), thus favoring the progression of alcoholic cardiomyopathy. Notably, a recent in vitro and in vivo study by Fan et al. [134] demonstrated that astaxanthin, a lutein carotenoid antioxidant, can protect against acute alcoholic cardiomyopathy by maintaining cardiac systolic function and decreasing pathological changes, OS, apoptosis, and inflammation [134]. Moreover, from in vivo and in vitro findings, it also appears that the haploinsufficiency of Beclin1, a well-known regulator of autophagy (see also below Section 6), enhances acute alcohol challenge-induced myocardial remodeling and contractile dysfunction via inhibition of ferroptosis, a kind of programmed cell death related to iron and characterized by an increase of lipid peroxides [135].

Acute kidney injury is a clinical syndrome characterized by rapid deterioration of renal function. Alcohol could exacerbate acute kidney injury by provoking inflammatory responses and OS, although this speculation is controversial and remains to be confirmed [14]. A recent study of Zhan and colleagues (2024) [14] established that alcohol ingestion aggravates acute kidney injury and exacerbates tubular cell damage and apoptosis in mice, supporting the idea that chronic alcohol consumption raises the susceptibility and severity of kidney disorders. Remarkably, this adverse effect of alcohol ingestion appears exclusively in female, but not male, mice, featuring a noteworthy gender difference in determining the vulnerability to alcohol toxicity. Interestingly, it is known that ALDH is more active in males than in females, whereas ADH activity is higher in female mice than in male mice. Hence, it is possible that after alcohol consumption, females accumulate more acetaldehyde, which is responsible for causing greater injury to the kidneys [14].

Although several studies propose that light or moderate alcohol consumption might reduce the risk of chronic kidney disease [136], others indicate that alcohol drinking might be correlated with the development and increased progression of chronic kidney disease [137]. Indeed, many experimental studies have indicated that alcohol consumption may damage renal tubular reabsorption and glomerular filtration [138]. Furthermore, alcohol intake interferes with kidney activity, leading to a reduction in the number of nephrons [139]. Moreover, patients with AUD often have rhabdomyolysis related to direct muscular tissue toxicity as a consequence of heavy, chronic alcohol consumption, as well as other insults, such as hypophosphatemia and hypokalaemia [140].

Chronic alcohol consumption negatively affects immune system homeostasis, and, predictably, individuals who consume large amounts of alcohol are at increased risk of acute pulmonary injury and infections. Indeed, chronic heavy alcohol exposure disrupts alveolar epithelial integrity and, by diminishing endogenous antioxidant levels, such as GSH, also decreases alveolar macrophage function [141]. In this condition, the cytotoxicity of acetaldehyde plays an important role in inducing OS and also modifying the stomach mucosal barrier, thus impacting the gut–lung axis [142]. Furthermore, alcohol ingestion is a risk factor for cancer. Acetaldehyde is one of the major causes of alcohol’s oncogenic effect, mostly through the production of acetaldehyde-derived DNA adducts [143]. Different studies provide support for a role of ADH and ALDH2 gene polymorphisms in alcohol drinking-related cancer. Remarkably, reduced ALDH2 expression in tumor tissue increases acetaldehyde concentrations available to stimulate the production of collagen in human fibroblasts, a hallmark of solid tumors [144], remodeling tumor architecture and impairing immune surveillance.

## 5. Role of Oxidative Metabolism in the Central Effects of Alcohol

### 5.1. Oxidative Metabolism: Dopamine, Reinforcement and Motivation

While there is no question that the mesolimbic dopamine (DA) system is critically involved in the reinforcing and motivational properties of alcohol [145,146], it is extensively documented that alcohol also engages many intracellular signaling mechanisms [147] and diverse neurotransmitter systems to exert its differential central effects. These include the GABAergic [148], glutamatergic [149], serotonergic [150], opioidergic [151], and endocannabinoid [152] transmissions, which are differentially responsible for the ability of alcohol to exert distinct behaviorally relevant effects, such as anxiolysis, locomotor stimulation, motor incoordination, sedation, and narcosis, as well as for supporting the expression of its DA-mediated reinforcing and motivational properties.

For the sake of clarity and to maintain the focus of the present work, it is critical to keep in mind a fundamental distinction between the effects that can be ascribed to alcohol “as such”, i.e., before being subjected to its oxidative metabolism, and those that can be attributed to acetaldehyde. Indeed, in the context of the present review, this distinction allows focusing on the significance that oxidative metabolism assumes with respect to the numerous and diverse aspects of alcohol effects. Briefly, as it is not the primary focus of this work, as far as the acute effects of alcohol “as such” are concerned, these rely mainly on its ability to modulate GABA transmission and to enhance GABAA-receptor-dependent functions [152,153,154], potentiating evoked inhibitory postsynaptic currents and vesicular GABA release [155]. Notably, both peripheral and central oxidative metabolism, i.e., both peripherally and centrally generated metabolites, may contribute similarly to the central effects of alcohol. Accordingly, the discussion of the literature will follow the main two lines of research aimed at characterizing the role of the oxidative metabolism of alcohol in its reinforcing and motivational effects: (i) the first one involves alcohol’s main metabolite, acetaldehyde, generated in the periphery and (ii) the second one depends on the efficiency of central enzymatic oxidative processes and hence implies, in the central effects of alcohol, a critical role of brain oxidative metabolism.

As far as the first line of this research is concerned, these studies were inspired by two main observations from the 1950s and from the 1970s. The first observation was that, while on disulfiram, some alcoholics could feel attracted to further alcohol consumption [156], and the second one was that circulating levels of acetaldehyde and “related” adducts were found in recently abstaining alcoholics [157]. Such evidence strongly suggested, on one hand, that acetaldehyde may play an additive role in the reinforcing and motivational properties of alcohol and, on the other hand, that acetaldehyde and “related” adducts could represent valuable markers of chronic alcohol consumption [157]. Interestingly, already at that time, these so-called “related” adducts were not simply being attributed the functional role of serving as useful markers of alcohol heavy intake, but they were also hypothesized to play some role in the mechanisms of alcohol addiction [157,158]. As a matter of fact, as we will discuss below, both acetaldehyde and “related” adducts were, after those pioneering and inspiring observations, explicitly proposed as the main chemicals responsible for alcohol reinforcing and motivational properties [159,160,161,162]. It is important to note, however, that this responsibility has to be limited to the acute reinforcing and motivational actions of alcohol (see for this issue the “first hit hypothesis” [146]). Moreover, at that time, a direct and unequivocal demonstration of their mechanism was yet to come [163].

The general hypothesis, generated by Chevens [156] and Cohen and Collins [157], proposed that acetaldehyde accumulation from peripheral metabolism might cross the enzymatic barrier of ALDH, which is also expressed at the blood–brain barrier [164,165], and reach brain targets mediating alcohol’s effects. Experiments testing this hypothesis have been summarized in seminal reviews [159,162,166,167,168], spanning over twenty years of studies assessing acetaldehyde’s central effects and its role in alcohol’s central actions. These studies also highlighted some controversies, particularly regarding the failure to reliably detect acetaldehyde in brain tissue after systemic alcohol or acetaldehyde administration [159]. As for the “related” adducts, the in vivo determination of acetaldehyde is critical for understanding both its pharmacological profile and its contribution to alcohol’s central effects. Acetaldehyde is highly reactive and has a short half-life that makes its detection in the brain difficult using conventional analytical methods. Recent reviews [169] suggest that non-invasive approaches, such as magnetic resonance spectroscopy or biosensors (devices combining biological components with physicochemical detection), could provide definitive tools to address this question [170]. High-throughput quantitation using gas chromatography/mass spectrometry with positive chemical ionization has demonstrated the feasibility of detecting alcohol and acetaldehyde under basal conditions and in the presence of ALDH inhibitors in rodent plasma, blood, serum, and organs [171,172]. Additionally, headspace gas chromatography coupled with selected-ion monitoring mass spectrometry now allows acetaldehyde determination in brain tissue [172,173].

Direct comparisons of alcohol and acetaldehyde effects after peripheral administration in mice and rats, using locomotor activity as a behavioral readout, showed that acetaldehyde, similarly to alcohol, can elicit stimulatory [174] or inhibitory [175] effects depending on dose and timing. Sedative, hypnotic [176], and potential anxiolytic effects [174,175] were also observed. Furthermore, both alcohol and acetaldehyde stimulate DA transmission [177] and indirect DA-dependent postsynaptic indices of neuronal activation in the mesolimbic system [178,179].

As far as the second line of research addressed in this review is concerned, this focused on the role of metabolism in the reinforcing and motivational effects of alcohol with respect, in particular, to the involvement of the DAergic system; the role of alcohol metabolites in its central effects has seen a profound revision by the demonstration that this might consistently take place in the brain by means of peroxisomal CATs [180,181]. This discovery contributed to profoundly revisiting the significance of centrally generated metabolites. In this context, the work of Drs. Aragon and Amit in the early 80s was particularly influential. In fact, beginning by disclosing, in rats, a positive correlation between brain CAT activity and voluntary alcohol consumption [182], these investigators also demonstrated, through the use of CAT inhibitors, such as 3-amino-1,2,4-triazole, the involvement of brain CAT (a) in voluntary alcohol consumption [183], (b) in determining the motivational properties of alcohol [184,185], but also (c) in alcohol-dependent motor activity [184] and (d) alcohol-induced narcosis, (e) lethality, and (f) hypothermia [186]. These studies also further supported the suggestion [187] that central CAT-mediated production of acetaldehyde could represent a possible mechanism for the expression of the psychopharmacological effects of alcohol. This hypothesis was later on prompted by a clever approach with shRNA to silence central CAT expression in the Ventral Tegmental Area (VTA) of alcohol naïve rats. These original studies, by the group of Dr. Israel, using lentiviral vectors coding for a shRNA designed to inhibit about 70 to 80% the synthesis of CAT [188], disclosed that such suppression could virtually abolish the voluntary consumption of alcohol [188,189,190]. Moreover, similar findings were also obtained by other groups that extended their investigations to the complementary role played by brain ALDH [191].

Almost in parallel, although at a slower pace and intensity, another line of research aimed at investigating the role of metabolism in the reinforcing and motivational effects of alcohol was that on the role of acetaldehyde’s “related” adducts. As mentioned above, this research path was prompted in the 1970s by the original observations by Collins and Colleagues [192], Davis and Walsh [158], and Yamanaka et al. [193] and was revitalized by a behavioral study reporting that salsolinol, the condensation product of acetaldehyde and DA [194], is endowed with reinforcing properties, as demonstrated by a place conditioning study in rats exposed to conditioned fear stress [195], a finding later on confirmed also by Quintanilla and Colleagues [196]. The original studies on this “related” adduct were, at that time, inspired by its structural similarity with morphine [197,198,199] and aimed at characterizing its pharmacological profile with respect to its claimed opioid-like profile, although failing to yield resolutive evidence [200]. However, and despite its reputation of being a substance with poor blood–brain barrier permeability [201], the interest in salsolinol increased significantly, and a relevant number of subsequent studies demonstrated that its local administration into the rat VTA, the region of origin of the mesolimbic DA system, elicits locomotor stimulant effects [202], conditioned place preference [203] and DA transmission in the nucleus Accumbens [203,204]. Interestingly, these studies also showed that the effects of local administration of salsolinol were mediated by opioid receptors since the application of µ-opioid receptor antagonists could prevent them [202,203]. Moreover, the role of µ-opioid receptors in the effects of salsolinol on DA neurons in the VTA was further supported by electrophysiological [205] and molecular modeling studies [206]. Further studies also disclosed that salsolinol is self-administered by rats [207] and affects alcohol consumption, as its repeated administration stereospecifically causes behavioral sensitization and facilitation of alcohol intake [196,208].

Thus, indirect evidence was accumulated to suggest the possibility that salsolinol could replicate many effects of alcohol specifically related to the activation of the DA mesolimbic system [160,204]. However, the evidence that salsolinol was indeed a molecule formed in vivo upon alcohol systemic administration or ingestion was still missing [163], and the attempts to prove its metabolic origin from systemic alcohol were unsuccessful [171,209].

**Figure 2:**
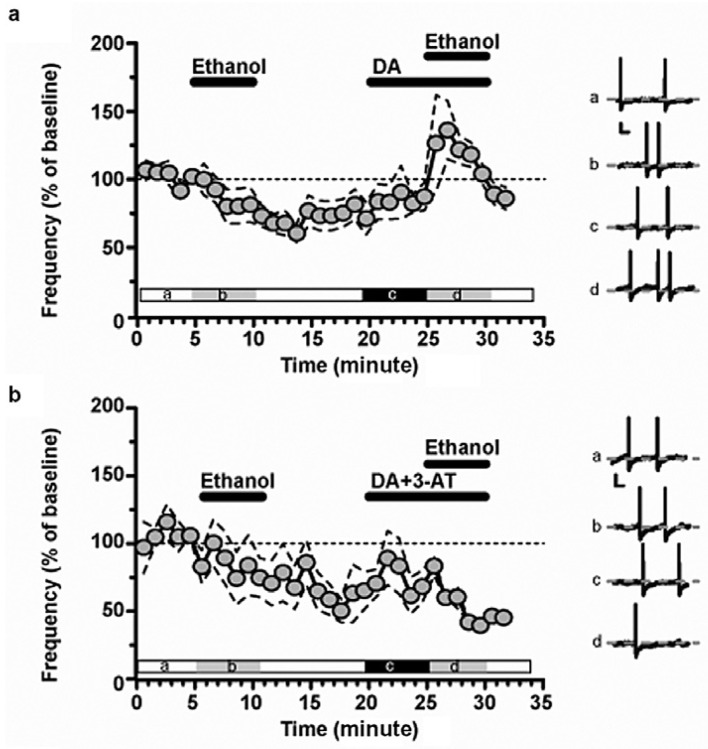
Alcohol (Ethanol)-induced excitation of dopamine (DA) neuronal firing rate requires its oxidation into acetaldehyde and the presence of DA. (**a**) Time course graph illustrating the averaged effects of alcohol (100 mM) on the firing rate of DA cells of the posterior Ventral Tegmental Area (pVTA) in mice pretreated with alpha-methyl-p-Tyrosine (αMpT). Note that the effect of alcohol is restored in the presence of exogenous DA (10 nM). On the right-hand side of the panel, representative traces of the spontaneous activity of a DA neuron during baseline (a), alcohol (b), DA (c), and alcohol co-applied with DA (d) of an αMpT-treated mouse. Calibration: 15 mV, 125 ms. (**b**) Time course graph illustrating the averaged effects of alcohol (100 mM) on the firing rate of DA cells of the pVTA in mice pretreated with αMpT. The effect of alcohol is abolished in the presence of DA (10 nM) when acetaldehyde formation is prevented by 3-amino-1,2,4-triazole (3-AT) application (1 mM). On the right-hand side of the panel, representative traces of the spontaneous activity of a DA neuron during baseline (a), alcohol (b), DA and 3-AT (c), and alcohol co-applied with DA and 3-AT (d) of a αMpT-treated mouse. Calibration: 15 mV, 125 ms. Data are normalized to the baseline. *n* = 6 for all groups. Reprinted/adapted with permission from Ref. [210]. 2013, M. Melis.

**Figure 3:**
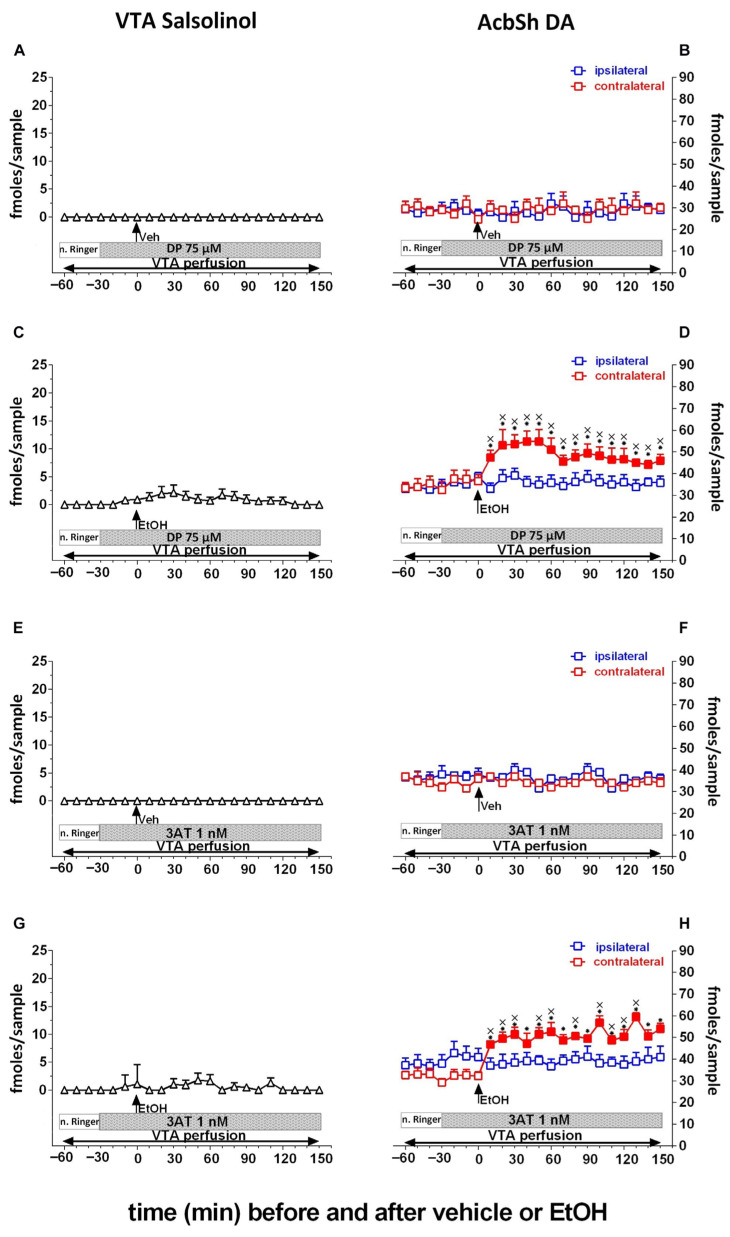
Effects of intragastric administration of vehicle (tap water, 10 mL/kg) [(**A**) n = 6, (**B**) n = 8, (**E**) n = 6, (**F**) n = 8] or alcohol (EtOH) (1 g/kg, 20% *v*/*v*) [(**C**) n = 11, (**D**) n = 8, (**G**) n = 11, (**H**) n = 9] in the presence of reverse dialysis application in the pVTA, beginning 30 min before EtOH administration, of D-penicillamine (DP) (75 mM) (**A**–**D**) or 3-amino-1,2,4-triazole (3AT) (1 nM) (**E**–**H**) on pVTA salsolinol (**A**,**C**,**E**,**G**) and on ipsilateral and contralateral nucleus accumbens shell (AcbSh) DA (**B**,**D**,**F**,**H**) dialysates. Horizontal bars depict the contents of the pVTA perfusion fluid during the experiments. Vertical arrows indicate the last pVTA or AcbSh microdialysis sample before vehicle or alcohol administration. Filled symbols indicate samples representing *p* < 0.001 vs. basal; * *p* < 0.01 vs. vehicle administration; ^×^ *p* < 0.01 vs. ipsilateral area. Reprinted/adapted with permission from Ref. [211]. 2021, V. Bassareo.

**Figure 4:**
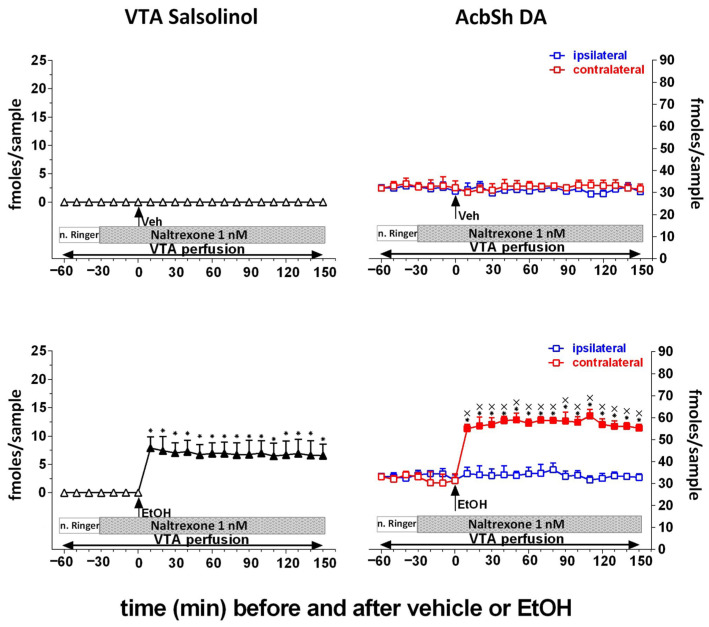
Effects of intragastric administration of vehicle (tap water, 10 mL/kg) [(**Top left**) n = 9, (**Top right**) n = 8] or alcohol (EtOH) (1 g/kg, 20% *v*/*v*) [(**Bottom left**) n = 12, (**Bottom right**) n = 11] in the presence of reverse dialysis application in the pVTA, beginning 30 min before alcohol administration, of naltrexone (1 nM) on VTA salsolinol (Left: Top and Bottom panels, respectively) and on ipsilateral and contralateral AcbSh DA (Right: Top and bottom panels, respectively) dialysates. Horizontal bars depict the contents of the pVTA perfusion fluid along the experiment. Vertical arrows indicate the last pVTA or AcbSh microdialysis sample before vehicle or alcohol administration. Filled symbols indicate samples representing *p* < 0.001 vs. basal; * *p* < 0.01 vs. vehicle administration; ^×^ *p* < 0.01 vs. ipsilateral area. Reprinted/adapted with permission from Ref. [211]. 2021, V. Bassareo.

This puzzling and critical question was answered firstly by an ex vivo study showing that salsolinol is formed in the VTA in the presence of alcohol only if DA is available and if the oxidative activity of CAT (Figure 2) is fully preserved [210]. Moreover, this evidence was robustly confirmed by an in vivo brain microdialysis study demonstrating (i) that salsolinol, otherwise not present, becomes detectable after an acute systemic administration of alcohol by gavage, (ii) that “this” salsolinol is necessary to trigger the release of DA neurotransmission in the nucleus Accumbens [211,212] (Figure 3), and (iii) that such stimulation of mesolimbic DA by alcohol-derived salsolinol is mediated by the stimulation of µ-opioid receptors in the VTA (Figure 4) [211].

### 5.2. Alcohol Chronic Intoxication: Oxidative Stress and Neuroinflammation

As thoroughly discussed, chronic alcohol intoxication is associated with the activation of central pro-oxidant mechanisms [9,213], as well as with alterations in the immune brain response [214]. Neuroinflammation is defined as the inflammatory response of the Central Nervous System (CNS) and comprises a plethora of responses mediated by resident glial cells in the CNS (microglia, oligodendrocytes, and astrocytes), non-glial resident myeloid cells (macrophages and dendritic cells), as well as peripheral leukocytes [215]. Inflammatory cells secrete reactive species that cause OS and that can further promote intracellular signaling cascades, hence leading to inflammation. Under such a vicious circle, neuroinflammation and OS can stimulate one another, especially in pathological conditions [216]. Accordingly, when redox cellular states are in balance, the inflammatory response acts as a defense mechanism, but in the case of a redox imbalance, such as upon binging or protracted alcohol consumption, the inflammatory response loses its protective function, generating neuroinflammation in the CNS and leading to progressive tissue damage.

Key pathways through which alcohol exerts its neuroinflammatory effects involve the activation of microglia [217] and astrocytes [218]. Although equipped with efficient antioxidant defense mechanisms, microglia are highly responsive to redox signaling, and ROS stimulate microglia-mediated production and release of inflammatory mediators [219]. Notably, activated microglia also produce ROS themselves, contributing to the generation of a self-sustaining perilous loop of inflammation and OS [220]. Similarly, astrogliosis, triggered by astrocytic response to injury, involves the release of pro-inflammatory cytokines, the enhancement of ROS production, as well as the impairment of glutamate homeostasis [221]. Intriguingly, the broad-spectrum brain-penetrant antibiotic minocycline prevents microglia activation, and, in mice, it has been reported to decrease voluntary alcohol intake and to attenuate withdrawal-induced anxiety and alcohol cue-induced relapse [222].

**Figure 5:**
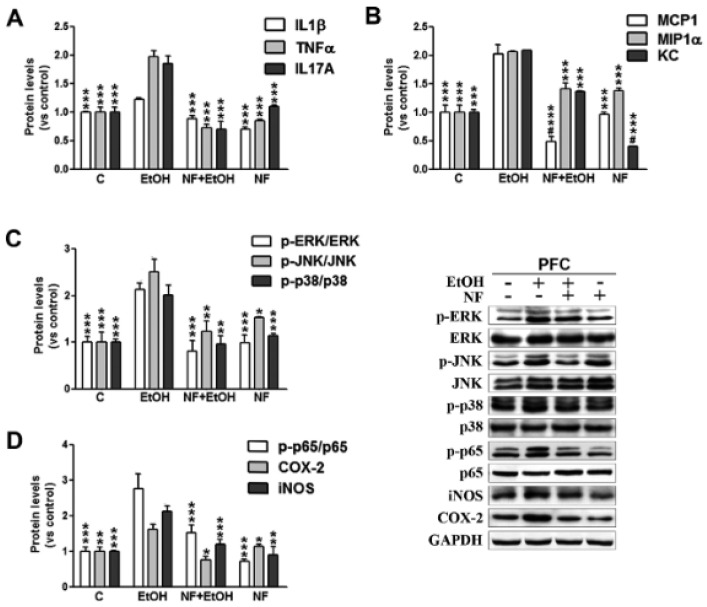
Nalmefene (NF) prevents the neuroinflammation induced by intermittent alcohol (EtOH) treatment in adolescence. The levels of (**A**) cytokines IL1β, TNFα, IL17A, and (**B**) chemokines MCP1, MIP1α, and KC in the prefrontal cortex (PFC) of the mice treated, or not, with EtOH or NF in adolescence. (**C**) The immunoblot analysis and quantification of the phosphorylation of MAPKs ERK, JNK, and p38, along with the (**D**) NFjB-p-p65, iNOS, and COX-2 levels in the PFC. A representative immunoblot of each protein is shown. GAPDH, or the total form of ERK, JNK, p38, or p65, was used as a loading control. Data represent mean SEM, n = 8 mice/group. * *p* < 0.05, ** *p* < 0.01, *** *p* < 0.001 compared to the EtOH-treated group, # *p* < 0.05 compared to the control group. Reprinted/adapted with permission from Ref. [228]. 2017, J. Montesinos.

Glial cells activation is also associated with increased expression of the chemokine monocyte chemoattractant protein-1 (MCP-1) [223,224]. Clinical evidence showed increased MCP-1 protein levels in the cerebrospinal fluid (CSF) of individuals with AUD [225] and in the VTA, substantia nigra, hippocampus, and amygdala, which are key regions of the reward system, of alcoholic brains compared to controls [223]. In alcohol-preferring rats, MCP-1 levels were found to be higher in the central amygdala and VTA, and inhibition of MCP-1 expression in these areas reduced alcohol intake [226]. Moreover, in a translational study, Lanquetin and colleagues [227] found elevated plasma levels of MCP-1, together with IL-8, TNF-α, and Macrophage Inflammatory Protein-1β (MIP-1β), in both humans with AUD and in selectively bred alcohol-preferring rats [227]. Interestingly, the European Medicines Agency (EMA) approved the AUD treatment nalmefene, a μ-opioid receptor antagonist, that also prevents the upregulation of MCP-1, IL-1β, TNF-α, and myelin damage induced by intermittent alcohol treatment [30,228] (Figure 5). Myelin injury is indeed a consequence of chronic neuroinflammation [229]. Prolonged alcohol exposure or exposure during critical developmental periods, such as the prenatal and adolescent stages, induces myelin developmental abnormalities that may underlie cognitive and motor deficits [230,231,232]. Strikingly, Guo and colleagues [233] demonstrated that even a mild level of alcohol exposure (5% alcohol for three weeks in adult mice) is sufficient to inhibit Oligodendrocyte Precursor Cells (OPC) differentiation and new myelin formation, suggesting that myelin genesis in adulthood is highly sensitive to alcohol.

In addition to minocycline and nalmefene, many other medications that reduce alcohol intake have been shown to target other OS-neuroinflammatory pathways [30], and new anti-inflammatory candidates are emerging [234]. For instance, the μ-opioid receptor antagonist and FDA-approved naltrexone also inhibits Toll Like Receptor-4 (TLR-4) downstream signaling; TPCA-1- or sulfasalazine-mediated inhibition of the redox-sensitive-pro-neuroinflammatory NF-κB signaling decreases alcohol intake and preference in mice. Finally, acute intraperitoneal administration of the antioxidant NAC reduces binge alcohol intake and reduces alcohol self-administration and relapse after abstinence in dependent rats [30].

Despite overwhelming evidence supporting the involvement of OS in multiple pathological dimensions of AUD and the encouraging results obtained in preclinical models, antioxidant interventions have often shown mixed and inconsistent clinical results [235,236,237,238]. This limited translational value is not unique to AUD but has also been observed in other diseases in which OS plays a prominent pathogenic role (e.g., neurodegeneration, cancer, etc.) [239]. Multiple biological, pharmacological, and methodological factors likely underlie these discrepancies. Preclinical studies typically administer antioxidants as a preventive measure or at the early stages of the disease, while in clinical practice, treatment usually begins after the establishment of the disease, when oxidative and inflammatory damage is already well established and less amenable to be reversed. Furthermore, differences in pharmacokinetics and dose scaling, as well as low bioavailability and nonspecific subcellular accumulation (e.g., insufficient mitochondrial targeting), might negatively affect the outcome of the treatment. Clinical heterogeneity further complicates the interpretation, as genetic polymorphisms in drug-metabolizing enzymes, differences in gut microbiota composition, as well as subjective differences in the baseline levels of OS or inflammation are rarely accounted for in trial design and could drastically contribute to the reported inconsistency [240]. These considerations underscore the need for improved delivery strategies, subcellular targeted compounds, and biomarker-guided patient stratification in future trials.

Despite the limitations and inconsistency observed in clinical trials, OS and neuroinflammation are regarded as interacting leading causes of elevated alcohol intake, tissue damage, and neurodegeneration. Alcohol may generate neuroinflammation by enhancing the production of oxidative species and triggering the release of pro-inflammatory cytokines, while, in parallel, enhanced neuroinflammation increases the redox imbalance, but also increases voluntary alcohol drinking, as well as withdrawal manifestations in rodents and humans [30], contributing to the formation of a complex vicious cycle composed of OS, neuroinflammation, and increased alcohol intake. The outcome of this cycle is alcohol-induced brain damage, which progressively leads to cognitive dysfunction. In this regard, the next paragraph will review how alcohol-dependent OS could be a risk factor for Alzheimer’s Disease (AD).

### 5.3. How Far Does It Go? Alcohol-Derived Oxidative Stress as Risk Factor for Alzheimer’s Disease

Chronic heavy alcohol exposure is increasingly recognized as an environmental factor that accelerates and exacerbates the pathological processes underlying AD. Preclinical [241,242,243] and epidemiologic [244,245,246] studies confirm that sustained excessive alcohol use can hasten the onset of AD and worsen its clinical signs. Indeed, emerging evidence indicates that multiple molecular pathways [247] and gene signatures [241,248] are shared between AUD and AD. One key mechanistic link between heavy drinking and AD pathology involves, again, OS and chronic inflammation, which are both drivers and outcomes of neurodegeneration in AD [249]. Mitochondria, as both sources and targets of ROS, as already stated, also lie at the center of this link. In AD, mitochondria often exhibit disrupted bioenergetics and impaired genomic maintenance, alongside abnormal mitochondrial biogenesis, imbalance in fission–fusion dynamics, and defective proteostasis. These structural and functional mitochondrial abnormalities play an essential role in AD pathogenesis [250]. Excessive alcohol exposure causes similar mitochondrial dysfunction: it impairs oxidative phosphorylation and perturbs the normal fission–fusion balance, thereby reducing mitochondrial membrane potential and calcium-buffering capacity [251,252]. Alcohol also suppresses the coactivator peroxisome proliferator-activated receptor gamma coactivator 1 alpha (PGC-1α) [253,254], a master regulator of mitochondrial biogenesis [255], hindering the replacement of damaged mitochondria. Additionally, and as previously discussed (see above Section 2.3), alcohol can directly damage mitochondrial DNA [251,256,257]; if unrepaired, this loss of mitochondrial genome integrity diminishes the production of vital mitochondrial proteins and further exacerbates organelle dysfunction. The resulting loss of mitochondrial function can trigger a vicious cycle, as accumulating ROS inflict even greater mitochondrial damage, leading to neuronal injury and potentially accelerating neurodegeneration in AD. OS derived from chronic heavy alcohol exposure also appears to worsen the hallmark proteinopathies of AD. The two classic neuropathological hallmarks of AD are neurofibrillary tangles composed of hyperphosphorylated tau protein and extracellular amyloid plaques composed of aggregated amyloid-beta (Aβ) peptides [258]. Alcohol has been reported to increase both tau phosphorylation and Aβ aggregation in the brain, and sustained OS critically fuels these aberrant changes. Mechanistically, chronic alcohol intake activates tau kinases, such as glycogen synthase kinase (GSK)-3B and JNK, while inhibiting tau-targeted phosphatase PP2A [243,247,259], a combination that directly increases tau hyperphosphorylation. Notably, OS itself can produce a similar effect by elevating GSK-3B and JNK and suppressing PP2A [260], thereby potentially linking an alcohol-induced oxidative environment to tau pathology. In parallel, alcohol increases Aβ accumulation by upregulating the amyloidogenic enzyme b-site APP cleaving enzyme 1 (BACE1) and impairing Aβ clearance via LDL-related Receptor Protein 1 (LRP1) [247,261]. These changes are also exacerbated by oxidative damage to these proteins. In addition to these pathways, alcohol-related OS may promote tau and Aβ pathology through increasing lipid peroxidation and through direct oxidative modifications. For instance, lipid peroxidation products generated during chronic OS can induce misfolding and aberrant conformational changes in tau, leading to its phosphorylation and polymerization [262,263,264]. These toxic by-products may also act upstream to amyloid pathology and contribute to impaired LRP1 function, further reducing Aβ clearance [265]. Moreover, tau protein is intrinsically susceptible to oxidation (particularly certain cysteine and methionine residues), which makes it more prone to aggregate [260]. Similarly, Aβ peptides can be oxidized at methionine-35, a modification known to promote the formation of Aβ dimers and oligomers [266]. In summary, chronic heavy alcohol intake initiates a cascade of OS and inflammatory insults, disrupting mitochondrial function and directly altering key proteins that might synergistically drive the neurodegenerative changes characteristic of AD.

## 6. Autophagy: A Cross-Cutting Integrative Mechanism of Alcohol-Dependent Oxidative Stress

Alcohol consumption profoundly affects cellular quality control mechanisms, particularly autophagy and mitophagy, which play a central role in maintaining mitochondrial integrity and limiting oxidative injury. Acute alcohol exposure can transiently activate autophagy, especially in the liver, as an adaptive response aimed at removing damaged proteins and organelles [267]. In contrast, chronic alcohol intake is associated with impaired regulation of autophagic pathways, leading to the accumulation of defective cellular components [267]. Mitochondria are major targets of alcohol-induced toxicity, as alcohol metabolism promotes OS, mitochondrial DNA damage, and bioenergetic dysfunction. Under physiological conditions, mitophagy selectively eliminates damaged mitochondria to preserve cellular homeostasis [268]; however, sustained alcohol exposure often compromises mitophagic efficiency, allowing dysfunctional mitochondria to persist and amplify ROS production [269]. This impaired mitochondrial quality control contributes to a vicious cycle of OS and cellular injury, with important implications for alcohol-related pathologies in metabolically active tissues, including the liver, brain, heart, and skeletal muscle [269].

Zhu et al. (2018) [270] reported that acute alcohol exposure in mice induces cardiac injury by triggering excessive autophagy in cardiomyocytes. This effect is mediated through OS-dependent activation of the JNK pathway, which leads to phosphorylation of Bcl-2 and disruption of the Bcl-2/Beclin-1 complex. The resulting increase in autophagy contributes to myocardial dysfunction and cell injury (see also above Section 4.2). Importantly, antioxidants, JNK inhibition, or suppression of autophagy attenuated alcohol-induced cardiac damage, indicating that the ROS–JNK–Bcl-2 signaling axis plays a central role in acute alcohol-related cardiotoxicity.

Recently, Ruiter-Lopez and colleagues (2025) [271] reviewed how excessive alcohol consumption harms the brain by causing OS and disrupting autophagy as a key cellular cleanup process. In the brain, alcohol is primarily metabolized by catalase and CYP2E1. As discussed (see above Section 2.2), such production of ROS and reactive nitrogen species by CYP2E1 leads to mitochondrial dysfunction and accumulation of damaged proteins, which impairs autophagy and mitophagy and contributes to neuroinflammation and neuronal death. Chronic alcohol exposure suppresses protective antioxidant pathways and exacerbates neurodegenerative changes. Moreover, this study highlights that the interaction between OS and autophagy regulation is complex and varies with drinking patterns.

Collectively, the evidence highlights autophagy and mitophagy as critical determinants of cellular resilience to alcohol-induced OS while revealing how their dysregulation contributes to tissue injury under excessive or chronic alcohol exposure. Acute alcohol intake may initially activate autophagic pathways as a compensatory mechanism to limit damage; however, sustained exposure disrupts these quality control systems, leading to the accumulation of dysfunctional mitochondria, increased OS, and progressive cellular injury. The resulting impairment of mitochondrial homeostasis establishes a self-perpetuating cycle of ROS production and bioenergetic failure that underlies alcohol-related damage in metabolically active organs, including the liver, heart, brain, and skeletal muscle.

Importantly, the impact of alcohol on autophagy and mitophagy is highly context-dependent, varying according to tissue type, exposure duration, and oxidative burden. As demonstrated in cardiac and neural tissues, excessive or aberrant activation of autophagy can be as detrimental as its suppression, contributing to cell dysfunction and death. These findings underscore the complexity of autophagy regulation in alcohol-related diseases and suggest that therapeutic strategies should aim to restore balanced mitochondrial quality control rather than simply enhancing or inhibiting autophagy. Targeting OS pathways, mitochondrial dysfunction, and their crosstalk with autophagic signaling may therefore represent promising avenues for mitigating alcohol-induced organ damage and preventing long-term pathological outcomes.

## 7. Conclusions

The evidence presented and discussed in the present review represents a set of complex phenomena arising from the multiple biological processes that a simple molecule—alcohol—can trigger through its oxidative metabolism.

Accordingly, under chronic alcohol exposure, the genetic variants of the enzymatic machinery involved in alcohol oxidative metabolism appear to be differentially responsible for a number of distinct consequences depending on the extent to which acetaldehyde and ROS interact with lipids, causing lipid peroxidation and generating additional reactive species, with proteins, modifying protein structure and function, and with DNA, inducing mutations.

Chronic alcohol consumption overwhelms cellular antioxidant systems and reprograms gene expression, including the Nrf2 signaling cascade, a master regulator of antioxidant defense. Downregulation of Nrf2-dependent enzymes, together with depletion of GSH and activation of pro-inflammatory NF-κB, perpetuates oxidative injury and sets the biochemical basis for alcohol-induced organ toxicity. Thus, alcohol oxidative metabolism promotes OS by negatively impacting cellular homeostatic processes responsible for mitochondrial energy production, lowering the availability of antioxidant species, and reducing the efficiency of antioxidant enzymes and mechanisms.

Moreover, by suppressing the synthesis of mtDNA-encoded protein subunits of the mitochondrial electron transport chain (ETC), chronic alcohol consumption dramatically impairs mitochondrial bioenergetics, leading to reduced ATP production, altered membrane potential, and compromised cellular energy homeostasis. In parallel, chronic alcohol exposure enhances pro-inflammatory gene expression, which amplifies oxidative injury, while ROS further induce pro-inflammatory cytokines. This cascade triggers a self-perpetuating cycle of OS and inflammation, contributing to the onset and progression of diverse pathological conditions, including liver injury, cardiac, pulmonary, and kidney failure, neurodegenerative disorders, and cancer.

Notably, OS intersects with epigenetic and epitranscriptomic regulatory layers, as alcohol-derived ROS alter DNA methylation, histone acetylation, miRNA networks, and RNA methylation through redox-sensitive enzymatic systems and one-carbon metabolism. Such modifications reshape chromatin accessibility and transcriptional output, contributing to tissue toxicity and, with more limited evidence, potentially to the motivational and reinforcing properties of alcohol.

## Data Availability

No new data were created or analyzed in this study. Data sharing is not applicable to this article.

## Abbreviations

AcbSh	Nucleus Accumbens Shell
AD	Alzheimer’s Disease
ADH	Alcohol Dehydrogenase
AH	Alcohol-associated Hepatitis
ALD	Alcoholic Liver Disease
ALDH	Aldehyde Dehydrogenase
AMPK	AMP-activated Protein Kinase
αMpT	Alpha-Methyl-P-Tyrosine
AREs	Antioxidant Response Elements
AUD	Alcohol Use Disorder
CAT	Catalase
cGAS	Cyclic GMP-AMP Synthase
CNS	Central Nervous System
CYP2E1	Cytochrome P450 2E1
DA	Dopamine
DNMTs	DNA Methyltransferases
ETC	Electron Transport Chain
FTO	Fat Mass and Obesity-associated Protein
GCL	Glutamate-Cysteine Ligase
GSH	Glutathione
GPx	GSH Peroxidase
GSK	Glycogen Synthase Kinase
H3K9ac	Histone H3 Acetylation at Lysine 9
HATs	Histone Acetyltransferases
HCC	Hepatocellular Carcinoma
HDACs	Histone Deacetylases
HO-1	Heme Oxygenase-1
Keap1	Kelch Like ECH Associated Protein 1
LRP1	LDL-related Receptor Protein 1
MAT	Methionine Adenosyl Transferase
MCP1	Monocyte Chemoattractant Protein-1
MDA	Malondialdehyde
MEOS	Microsomal Alcohol-oxidizing System
METTL3	Methyltransferase
m6A	N6-methyladenosine
MIP-1β	Macrophage Inflammatory Protein-1β
miRNAs	MicroRNAs
mtDNA	Mitochondrial DNA
NAC	N-acetylcysteine
NQO1	NAD(P)H:quinone Oxidoreductase 1
Nrf2	Nuclear Factor Erythroid 2-related Factor 2
OPCs	Oligodendrocyte Precursor Cells
OS	Oxidative Stress
PGC-1α	Peroxisome Proliferator-activated Receptor Gamma Coactivator 1 Alpha
PPAR	Peroxisome Proliferator-activated Receptor
ROS	Reactive Oxygen Species
SAH	S-adenosylhomocysteine
SAM	S-adenyl Methionine
SOD1	Superoxide Dismutase-1
TGFβ	Tumor Growth Factor Beta
TNFα	Tumor Necrosis Factor Alpha
VTA	Ventral Tegmental Area
YTHDF	YT521-B Homology Domain Containing Family
